# Meningococcal carriage within households in the African meningitis belt: A longitudinal pilot study

**DOI:** 10.1016/j.jinf.2017.11.006

**Published:** 2018-02

**Authors:** Nicole E. Basta, Abdoulaye Berthe, Mahamadou Keita, Uma Onwuchekwa, Boubou Tamboura, Awa Traore, Musa Hassan-King, Olivier Manigart, Maria Nascimento, James M. Stuart, Caroline Trotter, Jayne Blake, Anthony D. Carr, Stephen J. Gray, Lynne S. Newbold, Yangqing Deng, Julian Wolfson, M. Elizabeth Halloran, Brian Greenwood, Ray Borrow, Samba O. Sow

**Affiliations:** a Division of Epidemiology and Community Health, School of Public Health, University of Minnesota, Minneapolis, Minnesota 55454, USA; b Centre pour les Vaccins en Developpement-Mali, Centre National d'Appui a la lutte contre la Maladie (CNAM) Ministère de la Santé, Ex-Institut Marchoux, BP 251, Bamako, Mali; c London School of Hygiene & Tropical Medicine, Keppel Street, London, WC1E 7HT, United Kingdom; d Department of Veterinary Medicine, University of Cambridge, Cambridge, CB3 0ES, United Kingdom; e Meningococcal Reference Unit, Public Health England, Manchester Royal Infirmary, Manchester, M13 9WL, United Kingdom; f Division of Biostatistics, School of Public Health, University of Minnesota, Minneapolis, Minnesota 55454, USA; g Vaccine and Infectious Disease Division, Fred Hutchinson Cancer Research Center, Seattle, Washington 98109, USA; h Department of Biostatistics, University of Washington, Seattle, Washington 98195, USA

**Keywords:** Bacterial Meningitis, Meningococcal disease, *Neisseria meningitidis*, Carriers, Africa, Mali, Epidemiology

## Abstract

**Objectives:**

Carriers of *Neisseria meningitidis* are a key source of transmission. In the African meningitis belt, where risk of meningococcal disease is highest, a greater understanding of meningococcal carriage dynamics is needed.

**Methods:**

We randomly selected an age-stratified sample of 400 residents from 116 households in Bamako, Mali, and collected pharyngeal swabs in May 2010. A month later, we enrolled all 202 residents of 20 of these households (6 with known carriers) and collected swabs monthly for 6 months prior to MenAfriVac vaccine introduction and returned 10 months later to collect swabs monthly for 3 months. We used standard bacteriological methods to identify *N. meningitidis* carriers and fit hidden Markov models to assess acquisition and clearance overall and by sex and age.

**Results:**

During the cross-sectional study 5.0% of individuals (20/400) were carriers. During the longitudinal study, 73 carriage events were identified from 1422 swabs analyzed, and 16.3% of individuals (33/202) were identified as carriers at least once. The majority of isolates were non-groupable; no serogroup A carriers were identified.

**Conclusions:**

Our results suggest that the duration of carriage with any *N. meningitidis* averages 2.9 months and that males and children acquire and lose carriage more frequently in an urban setting in Mali. Our study informed the design of a larger study implemented in seven countries of the African meningitis belt.

## Highlights

• We demonstrated the feasibility of conducting a longitudinal, household-based study of meningococcal carriage in the African meningitis belt.
• During the cross-sectional screening visit, the carriage prevalence was 5% (20 carriers among 400 participants from 116 households).
• Over 17 months, 1422 swabs were obtained from 202 people in 20 households. 73 carrier isolates identified; 33 people (16.3%) carried at least once.
• Among all swabs collected, 84% of isolates were non-groupable, though 6 W and 9 Y isolates were identified. No A, C, or X carriers were found.
• The meningococcal carriage duration, any serogroup, was 2.9 months (95% CI: 1.6, 5.4). The acquisition rate was 2.3% per month (95% CI: 1.3, 3.8).

## Background

The African meningitis belt is an area of increased risk of bacterial meningitis characterized by distinct seasonal patterns in disease incidence with peaks in the dry season, large-scale epidemics every 5–12 years, and inter and intra-annual geographic variation.^1^, ^2^, ^3^, ^4^ This region, which stretches from Ethiopia in the east to Senegal and The Gambia in the west, has suffered high morbidity and mortality due to bacterial meningitis for more than a century, though rates have declined in recent years.^5^, ^6^, ^7^, ^8^ *N. meningitidis*, the bacterial pathogen primarily responsible for causing meningitis in this area, is transmitted person-to-person by close contact with the respiratory droplets or saliva of an infected person. The bacteria typically live in the pharyngeal passages of healthy humans without causing symptoms. However, following a 3–7 day incubation period, carriage with *N. meningitidis* can lead to invasive disease in some individuals, with high case fatality. Asymptomatic carriers are relatively common compared to cases of invasive meningococcal disease and are the primary source of transmission.^9^, ^10^

A review of meningococcal carriage in the African meningitis belt found that pharyngeal carriage ranged from 3% to 30% across several heterogeneous studies, that carriage is higher among contacts of cases than in the general population, and that carriage dynamics vary due to multiple context-specific factors, many of which are not fully understood.^11^ Despite knowledge of the key role carriers play in transmission, relatively little is known about the natural history of carriers, including the rate of acquisition and clearance of carriage, and the primary factors that lead from carriage to invasive disease. These characteristics are especially important in epidemiologic contexts where the incidence of invasive disease is high. A greater understanding of meningococcal carriage could provide insight into the epidemiology of meningococcal disease including the significant geographic and temporal variation observed in Africa and elsewhere.^1^ Investigating carriage could also help inform targeted strategies for reducing transmission, a top priority for meningococcal research.^12^, ^13^

Assessing changes in carriage requires longitudinal studies that monitor carriage status among the same individuals over time, yet most carriage studies in Africa have been cross-sectional. Repeated cross-sectional studies have provided significant insight, indicating that carriage varies little between seasons^14^ and that serogroup A carriage prevalence has declined even further following the introduction of the conjugate meningococcal serogroup A vaccine, MenAfriVac.^15^, ^16^, ^17^ Longitudinal studies among the same individuals, however, are more challenging because of the need for long-term follow-up, the burden of repeated swabbing of the pharyngeal passages to determine carrier status, and the low sensitivity of standard culture methods in detecting meningococcal carriage.^18^ Very few longitudinal studies of *N. meningitidis* carriage in Africa had been undertaken prior to this study. A study in Nigeria in the 1970s found that children were often the first identified carrier within a household and that the half-life of carriage was approximately three months.^19^ In a more recent study in Burkina Faso, the estimated average duration of carriage was shorter at 30 days.^20^ Additional empirical evidence could increase our understanding of transmission dynamics, inform the design of optimal vaccination programs to most effectively reduce transmission, and aid in planning strategies for preventing future outbreaks.

In 2010, prior to the launch of the MenAfriVac mass-vaccination campaigns across the African meningitis belt, the African Meningococcal Carriage Consortium (MenAfriCar) had a unique opportunity to investigate the natural history of carriage among the general population.^21^, ^22^ To take advantage of this opportunity, we conducted a longitudinal pilot study of carriage among residents of Bamako, Mali. Our pilot study was designed as a prelude to a larger and more complex longitudinal carriage study MenAfriCar conduced across seven countries of the meningitis belt.^23^

In this pilot study, we aimed to 1) standardize and test the implementation of clinical and laboratory protocols and 2) assess individual carriage status at multiple time points over an 18-month period. Our key considerations were to investigate how high retention in a longitudinal carriage study following entire households over more than a year would be and whether lab and field protocols would need to be revised to ensure high-quality data collection. Households were recruited for this study because of the close contact within families in this setting, suggesting that households are a relevant transmission unit. We aimed to estimate the rate of acquisition and clearance of carriage, the average duration of carriage, and whether carriage dynamics differed by sex and age.

## Materials and methods

### Study design

Community engagement meetings were held in Bamako, led by the Center for Vaccine Development-Mali (CVD-Mali), to provide information to and gain community support for these research activities from community leaders and representatives prior to the start of MenAfriCar carriage studies.

### Cross-sectional screening study

At the outset of our study, we conducted a cross-sectional, population-based, screening survey in the urban setting of the Djikoroni-para quartier in Bamako, Mali, in May 2010. We randomly selected households with at least two members from the existing demographic surveillance system (DSS) database developed and maintained by CVD-Mali. Within each household, we randomly selected up to five participants, one from each age group (<1 year, 1–4 years, 5–14 years, 15–29 years, and ≥30 years). We continued recruitment until 400 participants had been enrolled: 20 from the youngest age group; 80 from the second youngest group; and 100 from each of the remaining groups. Potential participants were eligible unless they had a serious acute or long-term illness.

### Longitudinal household follow-up study

Beginning in July 2010 one month after the cross-sectional study ended, we selected 20 households from the cross-sectional study and enrolled all household members (regardless of whether the individual has previously participated) into a follow-up study to identify changes in carrier status over the next 18 months. All household members from this subset of selected households were visited every four weeks July through November 2010 (six visits) by study teams. In December 2010, the MenAfriVac mass-vaccination campaign took place in Bamako; all residents aged 1–29 years were eligible for vaccination. When additional funding became available, household follow-up resumed 10 months later in September 2011 with visits conducted every four weeks through November 2011 (three visits).

### Sample and data collection

According to a standard protocol, at the cross-sectional study visit and the nine longitudinal study visits, study physicians collected a single pharyngeal swab using a sterile, dacron-tipped, plastic shaft swab by swabbing both the posterior pharynx behind the uvula and one *tonsillar fossa*.^24^ Pharyngeal swabs were plated immediately in the field on Thayer-Martin (TM) selective agar plates and returned to the laboratory within six hours. At each visit, participants (or their parents if they were under 15 years of age) were asked to provide responses to a survey, administered orally by trained field teams and designed to capture information including demographics and potential risk factors. At the initial cross-sectional visit, the head of the household or another adult also completed a questionnaire to report household characteristics including household size.

### Ethical considerations

A community consent meeting was held prior to study launch, as noted above. During study recruitment, the head of each selected household was asked to verbally confirm his or her agreement for the individual household members to be invited to participate. Individual participants aged 18 years and older and parents or guardians of younger children were asked to provide written consent after listening to the consent form read in Bambara, the local language. Those aged 12 to 17 years were asked to provide written assent and children less than 12 years old provided oral assent. The Ethics Committees of the University of Bamako Faculty of Medicine and the London School of Hygiene and Tropical Medicine and the Institutional Review Board (IRB) of the Fred Hutchinson Cancer Research Center approved the data collection and analysis. The University of Minnesota IRB also approved the data analysis.

### Laboratory analysis

The TM plates were incubated in 5% CO_2_ at 35–37 °C for up to 72 hours at the CVD-Mali lab. A single colony of typical morphology was selected, sub-cultured on a blood agar plate (BAP), and incubated for 18–24 hours in 5% CO_2_ at 35–37 °C prior to Gram staining and oxidase testing. The colonies remaining on the TM selective agar plate were collected with a sterile plastic loop, suspended in a cryotube containing 1 mL of Brain Heart Infusion (BHI) broth supplemented with 15% glycerol and stored at –80 °C as a back-up in case there was a need to go back to the original samples from study subjects.

Immediately following collection, identification of carrier isolates was undertaken on site using routine microbiological culture methods. After all sample collection was completed, confirmatory analyses indicated that some samples had been misclassified. Thus, all samples stored in the BHI broth from both the cross-sectional and longitudinal visits were re-analyzed at the Meningococcal Reference Unit at Public Health England (PHE) to determine *N. meningitidis* carrier status. At PHE, the frozen samples were thawed, sub-cultured onto selective gonococcal media (Oxoid, GCVCAT), and incubated at 37 °C in 5% CO_2_ for 18–24 and 48-hour review. Suspected *N. meningitidis* colonies were tested with oxidase reagents and Gram film. Any oxidase-positive, Gram-negative diplococci colonies were sub-cultured for serological identification, which was undertaken by initial screening by the dot-blot enzyme-linked immunosorbent assay (ELISA) (with National Institute for Biological Standards and Control monoclonal antibodies (NIBSC mAbs)) for serogroups A, B, C, Y, and W and/or additional and subsequent use of in-house polyclonal antibodies utilizing co-agglutination for serogroups B, C, Y, W, X, E, Z.^25^

An individual was defined as a positive carrier at a given visit if *N. meningitidis* was isolated by PHE from the swab collected at that visit. All results reported here were based on laboratory results analyzed at PHE using the microbiological and serological methods described above.

### Data analysis

Data were merged, cleaned and managed using STATA version 10 (StataCorp LP 2009) and analyzed using R version 3.3.0.^26^ Carriage prevalence was summarized by visit at the individual and household level. Individual time to acquisition and duration of carriage were estimated from a continuous time hidden Markov model (HMM) in R using the *msm* package.^27^ Due to our interest in assessing the natural history pre-MenAfriVac introduction and due to the 10-month gap between the follow up visits conducted in 2010 and 2011 during which carriage status was unknown, our primary analysis consisted of fitting the HMM to data from the May 2010 screening and first six monthly follow-up visits through November 2010. Time was defined as number of days from May 26, 2010, the first day of data collection during the cross-sectional screening survey. The HMM requires that initial values be supplied for certain parameters that are estimated during the fitting process: sensitivity and specificity were set to 0.8 and 0.9999 respectively; the initial probability of being a carrier was set equal to the sensitivity for carriers and to (1-specificity) for non-carriers; and the initial values in the transition matrix were estimated by the *msm* package. The initial values did not substantially affect the final estimates. We also assessed the acquisition rate per month and whether the duration of carriage (equivalently, the hazard ratios for acquisition and clearance) differed by sex and age group (<15 vs. ≥ 15 years old) by fitting HMMs including these covariates.

## Results

During the May 2010 cross-sectional study, 400 participants from 116 households were enrolled and assessed for carrier status. Participants ranged from 1 month to 83 years old and households ranged from 3 to 60 residents. In total, 5% of individuals from 18 (15.5%) households were identified as *N. meningitidis* carriers.

During the launch of the longitudinal follow-up study in July 2010, all 202 residents of a subset of 20 of the households that participated in the cross-sectional study were invited and enrolled. Based on PHE's analysis of the swabs, six of these households had at least one confirmed carrier identified during the previous cross-sectional study visit. Follow-up consisted of six monthly visits conducted in 2010 and three monthly visits conducted in 2011, resulting in 1422 swabs analyzed to determine carrier status during 9 visits conducted over 18 months. Table 1 describes characteristics of all participants in the cross-sectional study and of all participants enrolled in longitudinal follow-up. Table 2 summarizes participation, by visit. The proportion of participants attending each visit out of the total number of participants enrolled ranged from 100% (at the first follow-up visit) to 57.4% (at the eighth follow-up visit).

**Table 1:** A comparison of the characteristics of individuals and households that participated in the screening visit (May 2010) and the subsequent follow-up visits (beginning July 2010).

Individual	Cross-Sectional Screening Visit (n = 400)	First Longitudinal Follow-up Visit (n = 202)
	Mean (SD) or N (%)	Mean (SD) or N (%)
*Age*	19.30 (17.56)	22.01 (16.27)
*Sex (female)*	233 (58)	106 (52)
*Sore throat in past week*	17 (4)	5 (2)
*Cough in past week*	92 (23)	31 (15)
*Runny nose in past week*	133 (33)	57 (28)

Household	Screening visit (n = 116)	First follow-up visit (n = 20)
	Mean (SD) or N (%)	Mean (SD) or N (%)
*Total residents*	12.55 (10.09)	13.84 (5.78)
*Number of bedrooms*	3.98 (1.42)	3.52 (1.68)
*With at least one smoker*	51 (44)	7 (35)

**Table 2:** Proportion of participants attending each visit and proportion where carrier status was assessed for each of the longitudinal follow-up visits.

	Participants Attending Each Visit		Carrier Status Assessed	
	N	% of initial participants	N	% of visit participants
*Visit 1: Jul 2010*	202	100	201	99.5
*Visit 2: Jul/Aug 2010*	181	89.6	180	99.4
*Visit 3: Aug 2010*	179	88.6	177	98.9
*Visit 4: Sep/Oct 2010*	172	85.1	171	99.4
*Visit 5: Oct 2010*	166	82.2	165	99.4
*Visit 6: Nov 2010*	171	84.7	171	100
***Dec 2010/Jan 2011***	***————- MenAfriVac Campaign ————-***			
*Visit 7: Sep 2011*	126	62.4	126	100
*Visit 8: Oct 2011*	116	57.4	113	97.4
*Visit 9: Oct/Nov 2011*	118	58.4	118	100

**Figure 1:**
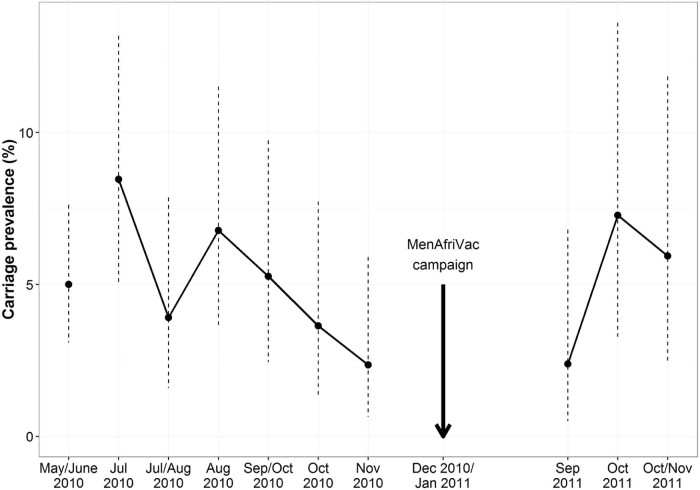
Individual *N. meningitidis* carriage prevalence observed during each study visit. Dashed vertical bars are 95% exact binomial confidence intervals calculated under the assumption that carriage is independent across individuals.

**Figure 2:**
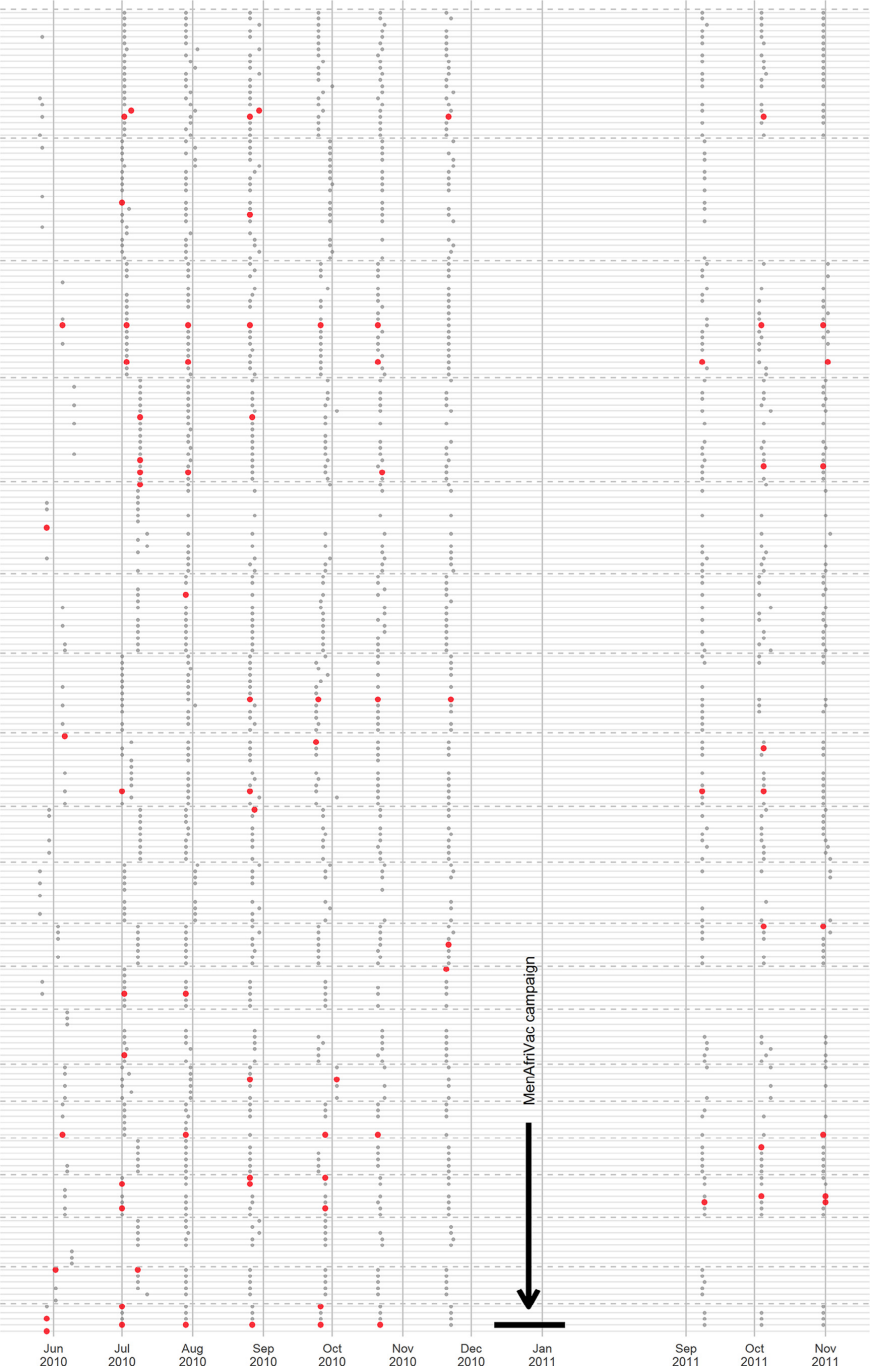
Changes in individual carriage status observed at each follow-up visit. The dashed horizontal lines separate the households. The gray dots represent the individuals within those households. Enlarged red points represent meningococcal carriers detected at that visit. Individuals maintain the same vertical position within their household from visit to visit, so persistence of carriage can be observed by following a single individual horizontally over time.

Among the 202 participants that attended at least one of the first six follow-up visits in 2010, 55 carriage events were identified from the 1063 swabs analyzed (5.2% of swabs positive). During this time, 13% of individuals (27/202) were positive for carriage at least once. Most of the positive carriers, 59% (16/27), were observed to carry at more than one visit. Among the 150 participants who continued to attend at least one of the three 2011 follow-up visits, 18 carriage events were identified from the 354 swabs analyzed (5.1% of swabs positive). During this time, 7.3% of individuals (11/150) were positive for carriage at least once. During all of the follow-up visits, carriage prevalence ranged from a high of 8.5% of participants during the first follow-up visit to a low of 2.3% during the seventh visit (Fig. 1). The percentage of households with at least one carrier ranged from a high of 55% during the first follow-up visit to a low of 16.7% during the seventh visit. The longest duration of carriage observed-i.e., the time period spanned by the longest string of consecutive monthly visits where an individual was identified as a carrier, including the screening visit, was 4.8 months. Changes in carriage status over time for all follow-up visits are shown in Fig. 2.

Based on the hidden Markov model fitted to the data from all swabs analyzed from the 202 individuals followed during the cross-sectional study plus the 6 follow-up visits in 2010, the acquisition rate per month was estimated to be 2.3 per 100 (95% CI: 1.3, 3.8). The mean duration of carriage was estimated to be 2.9 months (95% CI: 1.6, 5.4). For an individual carrier, the probability of clearing carriage within 30 days was 0.282 (95% CI: 0.169, 0.450). The model-based estimate of the sensitivity of the assay was 0.64 (95% CI: 0.49, 0.78) and the estimated specificity was very close to 1. The results of the hidden Markov model fitted to data collected during all study visits in 2010 and 2011 is given in Table S1. Acquisition and clearance rates were not significantly different between the first six follow-up visits and the three additional 2011 study visits (results not shown).

Table 3 presents the times and hazard ratios for carriage acquisition and clearance, by sex and age group. Males were estimated to have acquired carriage at approximately twice the rate of females (HR = 2.21 [95% CI: 0.66, 7.38]) and cleared carriage 66% more quickly (HR = 1.66 [95% CI: 0.51, 5.43]), but these differences are not statistically significant. A similar pattern was observed for individuals under age 15, who were estimated both to acquire and to clear carriage at approximately twice the rate of individuals aged 15 years and above, but these differences are also not statistically significant.

**Table 3:** A comparison of the time to acquisition and time to clearance of carriage by age and sex (along with 95% CIs), based on hidden Markov model results fitted to all data collected from the 202 participants from the 20 households followed during the cross-sectional survey plus the 6 follow-up visits in 2010 prior to MenAfriVac introduction.

	Hazard ratio for acquisition (95% CI)	Hazard ratio for clearance (95% CI)
Sex (Males vs. Females)	2.21 (0.66, 7.38)	1.66 (0.51, 5.43)
Age (≥15 vs. < 15 years)	0.41 (0.13, 1.32)	0.42 (0.13, 1.36)

	Time to acquisition in months (95% CI)	Time to clearance in months (95% CI)
Overall	36.0 (20.6, 63.0)	2.9 (1.6, 5.4)
Males < 15 years	16.1 (6.4, 40.1)	1.6 (0.7, 3.5)
Males ≥ 15 years	39.1 (14.3, 106.9)	3.9 (1.3, 11.5)
Females < 15 years	35.5 (11.4, 110.2)	2.7 (0.9, 8.0)
Females ≥ 15 years	86.3 (27.5, 270.5)	6.4 (1.7, 23.4)

From all swabs collected during the screening and all follow-up visits across the 18 months, we identified six serogroup W carrier isolates and nine serogroup Y carrier isolates. The majority of isolates were classified as non-groupable (78/93; 83.9% of isolates). Serogroup W was carried by four individuals, (one individual at the cross-sectional screening visit only, one individual during visit eight only, and two individuals who both carried during visits eight and nine). None were in the same household. Serogroup Y was carried by three individuals (one individual during the screening visit only, one individual during the screening visit and visits two, four, and five, and one individual during visits one, two, five, and nine). None were in the same household. No A, C, or X carriers were identified.

## Discussion

Our evidence suggests that the average duration of meningococcal carriage due to any serogroup in an urban setting in Mali, a hyperendemic country in the African meningitis belt, was 2.9 months, and that the estimated rate of acquisition of carriage was 2.3% per month. The vast majority of carrier isolates identified were non-groupable. Males and children may acquire and clear carriage more frequently than females and adults, respectively. Our study was conducted as an African Meningococcal Carriage (MenAfriCar) Consortium pilot study to inform a larger, multi-country study of transmission of meningococcal carriage within households. Our results are consistent with the estimated mean duration of carriage of 3.4 months (95% CI 2.7–4.4) reported in the main study^23^; both estimates are lower than the duration of carriage typically observed in European settings.^28^, ^29^, ^30^ The rate of acquisition we report is also similar to the estimated overall rate of acquisition of meningococci of 2.4% (95% CI 1.6–4.0) per month estimated in the main study.^23^

Our pilot study and the main multi-country MenAfriCar study differed substantially in the number of times each individual's carrier status was assessed, the time between the repeated assessment of carriage among the same individuals, and the overall length of time during which carriage was assessed. In our study, we assessed carriage monthly for 6 months then, following a 10-month gap, monthly for three months, identifying carriers over an 18-month period. In the main MenAfriCar study, carriage was assessed twice a month for two months and then monthly for four months, identifying carriers over a 6-month period. Yet, it is reassuring that results are remarkably consistent across the two studies.

Our analysis is limited in that we cannot be certain that individuals who were found to carry *N. meningitidis* across two or more consecutive visits were colonized by the same isolates. Many *N. meningitidis* isolates were non-groupable by serological methods, thus preventing us from assessing any differences by serogroup. Several previous studies have also identified a high proportion of non-groupable carriage isolates in samples collected in the African Meningitis Belt,^31^, ^32^ while others have not observed this trend.^15^, ^17^ While it is not clear why the proportion of unencapsulated meningococci is higher in this region than in other regions, one analysis suggested that air humidity may be correlated with acquisition of non-groupable carrier strains.^31^ Previous studies have estimated that a lower proportion of carrier isolates are non-groupable in non-hyperendemic regions such as Europe.^33^ Additional data and analyses are needed to evaluate possible causes for the predominance of non-groupable meningococci in a given geographic and temporal context. Understanding the descriptive epidemiology of non-groupable meningococci is a useful step towards generating hypotheses and examining possible drivers. In this regard, our study provides evidence for understanding carriage dynamics in the African meningitis belt, and is complementary to the findings of the larger MenAfriCar carriage study.^23^ In addition, we did not account for clustering by household in our model. Both the results of the pilot study we report here and the results of the main study highlight the richness of data that can be obtained through longitudinal studies, compared to cross-sectional studies alone.

Understanding the natural history of meningococcal carriage could have important implications for the prevention and control of meningococcal disease, especially in highly endemic areas. Our results and experience led to several lessons learned, including that confirming identification of carriers rapidly enough to enroll the entire household in follow-up can be challenging and that either additional efforts should be undertaken to prevent drop-out after a long period of study inactivity or that study duration should be kept as short as possible. These were central to informing the design and ensuring the success of longitudinal carriage studies subsequently undertaken in seven sites across the African meningitis belt by the MenAfriCar Consortium using a modified design. Our study was designed as a pilot study to demonstrate the feasibility of enrolling entire households in longitudinal follow-up to assess carrier status, a study design that has rarely been implemented. We found high participation rates across many months of follow-up and were able to identify and address key problems with the initial assessment of *N. meningitidis* carrier status. Pilot studies of complex clinical epidemiological designs are especially important in low-resource settings where considerable efforts may need to be undertaken to develop and refine protocols, test data collection and sample collection tools, and train staff prior to implementing larger, fully-powered studies.

## Funding

This research was funded by the Bill & Melinda Gates Foundation Grant 51251 (www.gatesfoundation.org), the Wellcome Trust Grant 086546 (www.wellcome.ac.uk), the US National Institutes of Health (NIH) grant R03AI092121, and the NIH Early Independence Award grant 1DP5OD009162 (Office of the Director and the National Institute of Dental and Craniofacial Research) (www.nih.gov). The funders had no role in study design, data collection and analysis, decision to publish, or preparation of the manuscript

## Conflict of interest

OM is currently working with his company MeniAfriCare for GSK. None of the other authors declared any potential conflicts.
