# Supplementary material for: Meningococcal carriage within households in the African meningitis belt: A longitudinal pilot study
Source: J Infect. 2018 Feb;76(2):140–8. doi: 10.1016/j.jinf.2017.11.006 (PMC5790055; doi:10.1016/j.jinf.2017.11.006)
Supplement: Appendix S1 — Supplementary material. [file mmc1.docx]

**Supplementary Appendix (S1): Results of the HMM model fit to data collected during all visits**

Based on the hidden Markov model fitted to data from all swabs analyzed from the 202 participants followed during the cross-sectional study visit plus both the six 2010 pre-MenAfriVac follow-up visits and the three 2011 post-MenAfriVac follow-up visits, the acquisition rate per month was estimated to 1.9% (95% CI: 1.1, 3.1). The mean duration of carriage was estimated to be 4.3 months (95% CI: 2.4, 7.5). For an individual carrier, the probability of clearing carriage within 30 days was 0.204 (95% CI: 0.122, 0.324). The model-based estimate of the sensitivity of the assay was 0.59 (95% CI: 0.45, 0.72) and the estimated specificity was very close to 1.

Table S1 presents the time and hazard ratio for carriage acquisition and clearance, by sex and age group. Males were estimated to have acquired carriage at approximately 1.4 times the rate of females (HR = 1.44 [95% CI: 0.47, 4.43]) and cleared carriage about 35% more quickly (HR = 1.35 [95% CI: 0.44, 4.08]), but these differences are not statistically significant. Individuals under age 15 were estimated to acquire infection approximately 5 times faster (95% CI: 1.7, 17.0) and clear infection at approximately 3 times the rate of individuals aged 15 years and above (95% CI: 1.0, 9.1).

**Table S1:** *A comparison of the time to acquisition and time to clearance of carriage by age and sex (along with the 95% CIs), based on hidden Markov model results fitted to all data collected from the 202 participants from the 20 households followed during the cross-sectional study visit plus both the six 2010 pre-MenAfriVac follow-up visits and the three 2011 post-MenAfriVac follow-up visits.*

|  | **Hazard ratio for acquisition**  **(95% CI)** | **Hazard ratio for clearance**  **(95% CI)** |
| --- | --- | --- |
| Sex (Male vs. Female) | 1.44 (0.47, 4.43) | 1.35 (0.44, 4.08) |
| Age (≥ 15 vs. < 15 years) | 0.19 (0.06, 0.60) | 0.34 (0.11, 1.04) |
|  |  |  |
|  | **Time to acquisition in months**  **(95% CI)** | **Time to clearance in months**  **(95% CI)** |
| Overall | 45.3 (27.3, 75.3) | 4.3 (2.4, 7.5) |
| Males < 15 years | 21.7 (9.2, 51.2) | 2.7 (1.2, 6.0) |
| Males ≥ 15 years | 115.1 (38.0, 348.9) | 8.0 (2.9, 22.0) |
| Females < 15 years | 31.3 (11.7, 83.3) | 3.6 (1.3, 10.1) |
| Females ≥ 15 years | 165.7 (56.4, 486.8) | 10.7 (3.6, 31.8) |
